# MTA apical barrier: *In vitro* study of the use of ultrasonic vibration 

**DOI:** 10.4317/jced.53085

**Published:** 2016-07-01

**Authors:** Begona Escribano-Escrivá, Pedro Micó-Muñoz, Alberto Manzano-Saiz, Teresa Giner-Lluesma, Nicolás Collado-Castellanos, Susana Muwaquet-Rodríguez

**Affiliations:** 1 Associate professor. Faculty of Health Sciences. Department of Dentistry. European University of Valencia. Spain; 2 Titular professor. Faculty of Health Sciences. Department of Endodontics and Conservative Dentistry. European University of Valencia. Spain

## Abstract

**Background:**

The apexification is the first alternative treatment on a permanent tooth when, after a tooth trauma and in the presence of immature apex trauma, pulp necrosis occurs. Many studies have demonstrated the efficacy of mineral trioxide aggregate (MTA) as apical sealing material of choice in these cases, but has a degree of filtration as all other materials. The objective of this study was to analyze the seal ability of MTA on the duct walls in immature teeth unirradicular apexes, using indirect vibration.

**Material and Methods:**

The study was conducted on 45 teeth divided into 3 groups: Group A or control group in which no vibration for placing the MTA was used, Group B and C or groups where indirect vibration analysis was used. All samples were immersed in methylene blue to assess filtration. After performing longitudinal cuts millimetric measuring were made of the degree of filtration, divided into 3 degrees (2mm each grade filtration).

**Results:**

Results obtained confirm our hypothesis, obtaining lesser degree of filtration those groups in which indirect vibration (Groups B and C) was performed. It was shown that the degree of filtration is closely linked to the degree of adaptation.

**Conclusions:**

MTA vibration offers better results in its adaptation to the canal walls, significantly reducing the degree of filtration.

** Key words:**Apexification, MTA, filtration, indirect vibration.

## Introduction

According to the AAE (American Association of Endodontics), the apexification is a method that induces the formation of a calcified barrier in a permanent tooth with open apex and necrotic pulp. One of the materials used for this treatment is calcium hydroxide, which also works well as pulp protector, direct and indirect pulp capping, intracanal medication, apicogénesis and irrigating solution (1). But despite the successes achieved with calcium hydroxide, technical apexification with this material has several disadvantages such as multiple appointments necessary over a long period of time, the unpredictable result of the formation of an apical barrier, and above all, susceptibility to coronary microleakage and fractures of these teeth so weakened (2).

The use of thermoplastic techniques are those that seal three-dimensionally the root canal systems. In cases apexification are not useful because of poor determination of a good apical stop, lack of sealing and the risk of overfilling exists (3).

Torabinejad M. describes the first case apexification with a type of material, called MTA (Mineral Trioxide Aggregate) (4). It has many indications among which is apexification. The MTA is a very alkaline cement with a pH of 12.5, with a low compressive strength, poor solubility and greater radiopacity than dentin. In addition, the MTA has demonstrated good biocompatibility with excellent sealed by their good marginal adaptation that reduces bacteria microfiltration. The pH obtained by the MTA after mixing is 10.2 and at 3 hours, is stabilized at 12.5. This pH similar to that of calcium hydroxide can also allow antibacterial effects. At the same time, if this substance is applied as apical sealing material probably this pH can induce hard tissue formation (4). 

The objective of this study was to assess “*in vitro*” degree of filtration of MTA in the walls of canals in unirradiculars permanent teeth with immature apices, when placed using indirect vibration against the conventional manner.

## Material and Methods

60 recently extracted single-rooted teeth were used, they were previously selected by radiologic examination to rule out internal resorptions, more than a canal, root caries, etc.

After being stored in 10% formaldehyde to the study, we proceeded to standardize the samples to a working length. For this diamond blades were used and samples proceeded to shorten coronal level discarding the crown, as well as the cutting of the last 3 millimeters to eliminate possible apical deltas that could interfere with the study. Once set a length of 16mm proceeded to the instrumentation of the canals. They were permeabilized with limes K-File # 10 and # 15 (DenstplyMaillefer Switzerland) and deeply irrigated with sodium hypochlorite 2.5% Monojet 27G needle to working length. Instrumentation by the system GT files Accessory 40/12%, 70/12%, 90/12% (DenstplyMaillefer Switzerland) actuated with an X-Smart motor (DenstplyMaillefer Switzerland) at the recommended speed by the manufacturer (800rpm). The last file was introduced by Coronal till it look over the apex, and again introduced by apical to create the apical divergence that characterizes teeth with immature apices.

Later a double layer of nail varnish were applied on the tooth surface of the samples, except for the apical surface, in order to allow leakage of dye through the interface material-tooth MTA. The MTA employee was Angelus® MTA (Angelus, Londrina, PR, Brazil) and placed with an MTA syringe holder from coronal to apical, compacting with plugger of Buchanan # 2.

The samples were divided into 3 groups with 20 samples each.

Group A (control): samples sealed with 4mm plug MTA without any indirect vibration.

Group B: samples sealed with of 4mm plug MTA in a single indirect increase to 4mm vibration.

 Group C: samples sealed with 4mm plug MTA with indirect vibration and 2 mm increments. First the syringe was introduced from coronal to apical 2mm, compacting with plugger and then the syringe was introduced 4mm and the last 2mm were sealed.

Indirect vibration was performed with the P5 Booster ultrasound unit with ultrasonic tip Satelec®, contact the Buchanan plugger at low power (power 4). The remains of MTA on coronal walls to 4mm of apical plug were cleaned with hand files, K-File # 50 and slightly moistened paper points. In contact with the MTA cotton swab dipped in distilled water was placed, as indicated by the manufacturer, to facilitate the setting of the material.

Once created the apical plug of MTA, all samples were sealed with sealing system injected thermoplastic technique, Pistol Obtura II (Obtura Spartan, Fenton, MO) with a 23G needle to get the absolute filling ducts. Once radiographically proven the absence of an interface between the MTA and gutta percha obturation, the coronal part was sealed with a simple composite restoration with the prior etching with orthophosphoric acid and adhesive placement. Samples were immersed in liquid methylene blue (Metiltionina chloride) for 48 hours to facilitate filtration, if any (5).

After 48 hours, the samples were rinsed under water pressure and dried. A longitudinal corono apical section using diamond disks to each were performed. In the case where the cut was not sufficiently focused sample was discarded. In total, and to simplify the results, 15 samples per group were used, with a total of 45 teeth. All samples were photographed using a Canon camera with 8.1V DC Ultrasonic Macro Lens Canon EF 100mm 1: 2.8 USM, Ø58mm. Then it checked by using millimeter measurement tools CS6 Photoshop program, the degree filtration.

**Figure 1:**
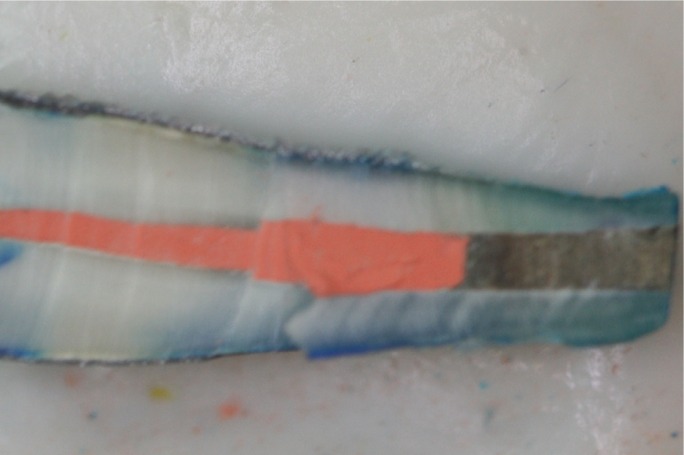
Specimen with leakage grade 1.

3 Grades were established:

Grade 1: 1-2 mm filtration (Fig. 1).

**Figure 2:**
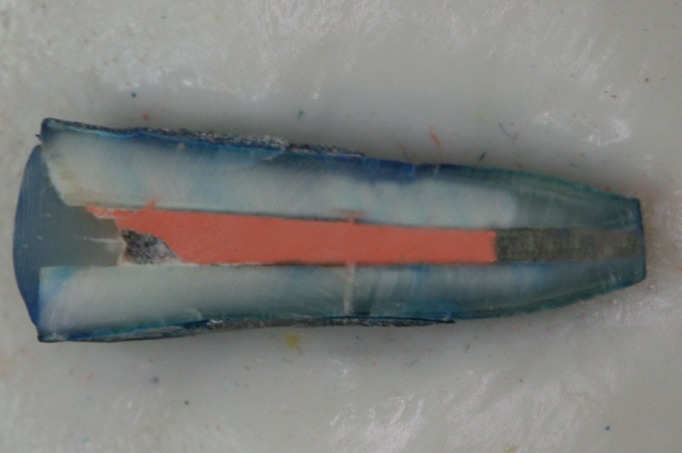
Specimen with leakage grade 2.

Grade 2: 3-4 mm filtration (Fig. 2).

**Figure 3:**
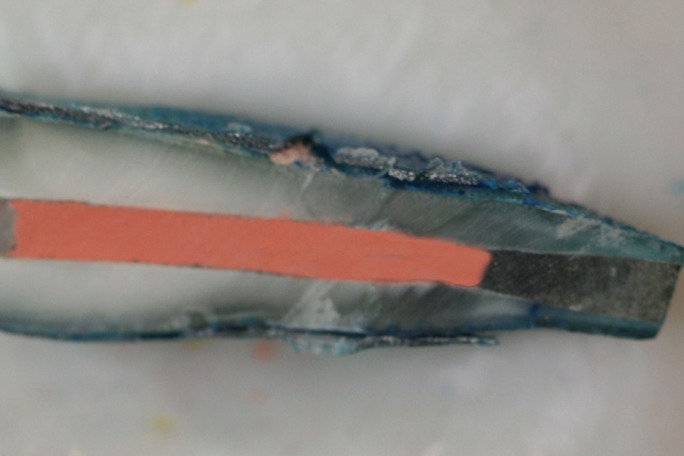
Specimen with leakage grade 3.

Grade 3: 5-6 mm or more filtration (Fig. 3).

Processing and data analysis was performed using SPSS 15.0 statistical package. The qualitative variables were presented with their frequency distribution. the association between qualitative variables was evaluated with the test chi-square χ2 or Fisher’s exact test, in the event that more than 25% of expected were under 5. For all tests a value of significance was accepted from 5%.

## Results

The following results were obtained:

Group A: 4 filtered through grade 1, 4 leaked to grade 2 and 7 filtered to grade 3.

Group B: 10 filtered through grade 1, 3 leaked to grade 2 and 2 filtered to grade 3.

Group C: 12 filtered through grade 1, 2 leaked to grade 2 to and 1 filtered to grade 3.

Statistically globally significant differences were observed in the distribution of filtering rate between the three groups of teeth. When analyzing two to two statistically significant differences between groups A and C. When comparing the group A versus B differences touched the level of statistical significance was obtained ( Table 1).

**Table 1:**
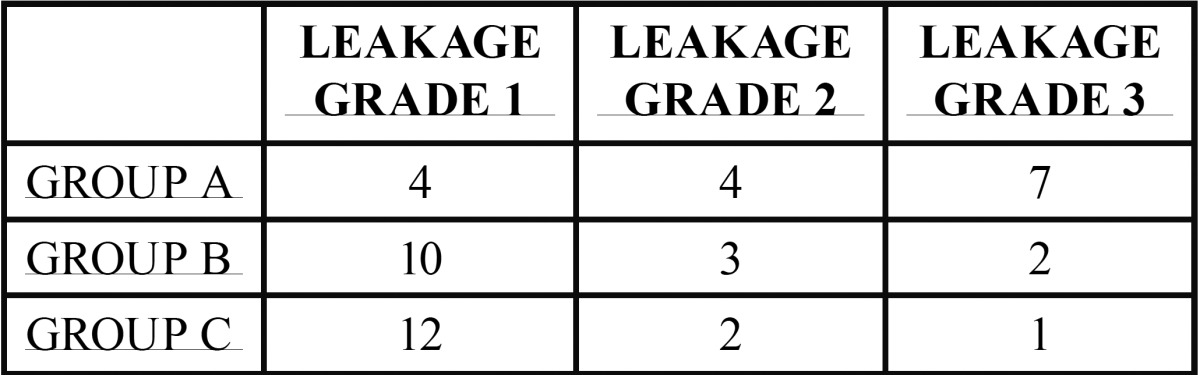
Number of specimens per grade of leakage.

## Discussion

Endodontic vibration is an issue that is currently booming in this branch of dentistry. There are already several years that the literature speaks of the vibration of the irrigant to enhance their properties as well as the effectiveness of cleaning, that after all, is one of the main objectives in the endodontic treatment (6,7). However, at the stage of apical sealing there are few studies using this technique.

Recent research (8) they have found that the addition of mineral trioxide induces the formation of apical tissue and its use is associated with less inflammation in the area, when compared with other materials. Based on the results of studies of the material used as sealing “retro” and in repairing perforations, the MTA is probably capable of activating cementoblasts to produce the matrix of the formation of cement (9). Possibly, this is due to their sealing ability, high pH, and / or the release of substances that activate cementoblasts to form a matrix for cementogenesis.

This study aims to evaluate the degree of marginal leakage in the canal walls when placed apical MTA as sealing material in permanent teeth with immature apices for the treatment of apexification. For this, they were simulated in mature tooth, immature apex by combining a orthograde and retrograde instrumentation. As recommended by the manufacturer, it must be placed between 3 and 5 mm of material to create the apical plug and placing increments.

We chose to give a thickness of 4mm to 2mm increments, so you can vibrate both increases, as in the case of Group C. A very similar to our study, comparing the degree of filtering white MTA to gray MTA they concluded that the leak was less in those cases where the MTA was vibrated and the thickness of MTA was about 5mm. Where a 2mm MTA plug, filtration was significantly higher (5). This suggests that it is not only important vibration, if not the number of millimeters thick of material placed.

Gianluca Plotinus and colleagues conducted a literature review on the use that could be given to the action of ultrasound in endodontics. He listed a number of indications such as get a straight access to the entrance of the Canals, locating calcified canals, remove pulp stones, remove broken instruments, posts and silver tips and increasing the action of irrigating. They have also been used for the condensation of the gutta-percha, conducting cavity “retro” in periapical surgery and for apexfixing the MTA in immature teeth apexes (10).

Witherspoon and Ham describe the use of ultrasound as an aid for positioning MTA. Irregularities and the divergent anatomy of some open apices can predispose small air bubbles from forming and trapping the material. It was demonstrated, as in our study, that by using ultrasonic vibration is the MTA improved sealing achieved. They also checked to find fewer radiographic gaps (11).

This study contradicts that of Aminoshariae *et al.* in which it is shown that a vibration of the material is not necessary for a better adaptation to the walls, and that the material of condensate obtained better results (12). Their findings could be due to his studies were performed in polypropylene tubes and not on human teeth. Furthermore, its analysis was limited to a superficial view of materials and internal voids could have passed easily unobserved. In their study he used direct activation of ultrasound, while in our indirect activation was used. In our study, the ultrasonic tip is not used directly to activate the MTA because even the smallest ultrasonic tip can not extend to the full length of the canal.

However, our study overlaps with that done by Lawley *et al.* in which they evaluated the ability of bacterial filtration teeth with apical barrier created with MTA and placed by vibration versus non-vibration, better results and lower rate of bacterial filtration in cases of vibration MTA, placing no increase (13). In their study only the last increase was vibrated MTA. In our study, the MTA was placed in layers, and vibration was also compared by layers obtaining the best results that way.

In another study, they assessed the density of MTA in two situations, manual apical plug condensation and condensation with indirect ultrasonic vibration. With only 1 second of vibration, they got better results in terms of adaptation of the material and fewer gaps in the duct (14).

Ruddle as in our study also recommends the use of indirect vibration. So after entering the MTA, contacts hand files K-File # 15 or # 20 to 1-2mm of the working length with an ultrasonic unit to produce such indirect vibration, this technique initially recommended to place the MTA in open divergent apices, but can also be used to place the MTA in drilling, and particularly cameral drilling floor (15).

## Conclusions

Indirect vibration on the MTA in open apices teeth get better results and statistically significant in terms of filtration degree than those in which said vibration is not performed.

Placing the MTA layered together with the use of indirect vibration improves sealing and this statistically significant result compared to the control group.

The use of vibration layered sealing get better result than the use of vibration mono-block, although this difference is not significant.
